# 

**Published:** 1915-02-06

**Authors:** 


### Honour for a V.A.D. Director.

The King has sanctioned the promotion in the Order of the Hospital of St. John of Jerusalem in England of Mr. Arthur William Faire, County Director of the Voluntary Aid Detachments of Leicestershire, as Esquire from Honorary Associate.

### Public Pharmacists' Annual Meeting.

"A HOSPITAL SECTION."

The annual general meeting of the Public Pharmacists' and Dispensers' Association was held on January 27 at St. Bride Institute, Ludgate Circus, E.C.

Mr. J. Hassall France occupied the chair, and was supported by a number of prominent institutional pharmacists, including Messrs. Kinsman, Thomas, Lindsey, Lindsay, Miller, Howell, Moore, Wolstenholme, Gibbons, Jenkin, Welford, Gibson (hon. sec.), etc.

The Council presented their annual report, which showed that in arranging instructive lectures, demonstrations, and discussions much useful work had been performed. It was noted with satisfaction that Mr. T. H. W. Idris, J.P., had again consented to occupy the presidential chair. The National Health Insurance Act has the close attention of the special committee appointed to watch any amendments or developments likely to prejudice the positions of institutional pharmacists. The attempt to secure direct representation on the Pharmaceutical Council is also mentioned in the report, and pharmacists in institutions are strongly advised to become members of the parent society, and thus qualify for a vote, not omitting to register this in an appropriate way. Although the candidate did not secure election, the number of votes polled gave ample justification for the support given to him by the Association. The treasurer's report showed a satisfactory state of the financial position.

The new officers for 1915 elected were :—President, Mr. T. H. W. Idris, J.P.; chairman, Mr. J. Hassall France; vice-chairman, Mr. R. Welford; hon. secretary and treasurer, Mr. Geo. W. Gibson; hon. secretary Poor-Law section, Mr. R. W. Lindsey; Council, Messrs. A. Howell, A. Jenkin, A. J. Gibbons, W. H. Windmill, F. W. Moore, W. E. Kinsman, H. Hewitt, and H. Skinner. The Council thus comprises pharmacists engaged in general hospitals, mental, Poor-Law, prison, and other public services.

The hon. treasurer having resigned, the Council recommended that the posts of hon. secretary and treasurer should be combined. This was endorsed by the members present.

The Chairman mentioned the fact that many pharmacists engaged in general hospitals did not trouble about " combining," but rather trusted to improve their position by individual application to their superiors.

Mr. France suggested that it might be feasible to have a " hospital section," to deal specially with matters concerning dispensing in general hospitals, just as the persons engaged in dispensing under Poor-Law authorities had a " section." Several of the older members and two of the original founders of the Association spoke hopefully of the future for public pharmacists, and the statement was made that the record of work already accomplished amply justified the foundation of this body of institutional workers.

A collection was made at the close for the " Belgian Doctors' and Pharmacists' Fund," and this will be forwarded, with any other amounts received by post, to the proper quarter by the hon. secretary.

Rates of Subscription : Twelve months, 6/6 ; Six months, 4/- ; Three months, 2/-, post free to any address in the United Kingdom. Abroad, Twelve months, 8/8; Six months, 5/-; Three months, 2/6, post free.—Address The Subscription Dept., "The Hospital," The Hospital Building, 28/29 Southampton Street, Strand, London, W.C.

